# A Rare Complication of Leptospirosis: Weil's Disease Diagnosed in the United States

**DOI:** 10.7759/cureus.43620

**Published:** 2023-08-17

**Authors:** Hinal Rathi, Aesha Patel, Rafik Elbeblawy, Ali Hassoun

**Affiliations:** 1 Internal Medicine, University of Alabama at Birmingham, Huntsville, USA; 2 Infectious Disease, Huntsville Hospital, Huntsville, USA

**Keywords:** leptospira interrogans, leptospirosis, leptospirosis with severe clinical manifestation, sepsis, weil's disease

## Abstract

Leptospirosis is mostly found in tropical regions such as Latin America and Southeast Asia. Here we present a case of leptospirosis in the United States in a 43-year-old African American male who had complications such as sepsis, acute renal failure, hyperbilirubinemia, and transaminitis. Through this case report, we want to highlight the rare occurrence of this infection in the United States. It should be considered high in the list of differential diagnoses for patients traveling to underdeveloped countries and participating in adventure sports. Early recognition and treatment of leptospirosis are essential in decreasing life-threatening complications.

## Introduction

Leptospirosis is a zoonotic infection caused by *Leptospira interrogans,* primarily found in underdeveloped tropic and subtropic countries. The Centers for Disease Control and Prevention has confirmed 23 total cases of this condition in the United States in 2022, while globally, around one million cases are diagnosed each year [1, 2]. Such rarity of leptospirosis in the United States makes the differential diagnosis of this infection not the first choice in patients, especially with the non-specific symptoms associated with the disease. Leptospirosis is transmitted through the urine of rats and via contaminated freshwater [1]. Agricultural workers, people living in poor living conditions (i.e., lack of proper shelter and sanitation), and people participating in water activities have a higher risk [1, 3]. It is a biphasic disease; the first phase lasts three to seven days with symptoms such as fever, conjunctival suffusion, rash, myalgias, headache, meningitis, and uveitis [4, 5]. In the second phase, immunoglobulin M (IgM) antibodies are produced and last 4-30 days or longer [4, 5]. It is in this phase that in 10% of such patients, it can progress to Weil's disease (WD), which manifests with jaundice, hemorrhage, renal failure, and myocarditis. The mortality rate is about 20% and up to 50% in pulmonary hemorrhage syndrome [5]. Diagnosis can be established through cultures (blood, cerebrospinal fluid, or urine), microscopic agglutination for antibody detection, and polymerase chain reaction tests. Treatment can include 7-10 days of tetracyclines, macrolides, ceftriaxone, or penicillin [5].

## Case presentation

A 43-year-old male with a history of migraines presented with complaints of nausea, vomiting, and fever. The patient reported recent travel to Jamaica and 10 days after his return to the US, the patient developed a fever of 103 degrees Fahrenheit, headache, chest discomfort, and burning sensation on his skin. During his visit to Jamaica, he traveled in a dune buggy, played in mud pits, jumped into the water from a 10-foot-high platform, and had mosquito bites. The patient also went to a different ER a week ago before his current admission and was provided symptomatic management and sent home. However, his symptoms progressively worsened with dark urine, muscle soreness, and intermittent episodes of difficulty breathing. His review of systems was positive for fever, chills, nausea, vomiting, intermittent dyspnea, cough, chest, abdominal and joint pain. He had a temperature of 100.1 degrees Fahrenheit, heart rate of 101 beats per minute, respiratory rate of 24, blood pressure of 133/69mmHg, and SpO2 of 99% on room air. Physical exam was remarkable for icterus and mild conjunctival suffusion. Labs indicated acute kidney injury, hyperbilirubinemia, mild transaminitis, elevated lipase, thrombocytopenia, rhabdomyolysis, and sepsis (Table 1).

**Table 1:** Abnormal lab findings

Lab study type	Lab name	Patient value	Reference range
Comprehensive metabolic panel	Sodium	125 mEq/L	136-144 mEq/L
	Anion Gap	17.6 mEq/L	4-12 mEq/L
	Glucose	345 mg/dL	64-100 mg/dL
	Blood urea nitrogen	40 mg/dL	6-20 mg/dL
	Creatinine	4.1 mg/dL	0.8-1.2 mg/dL
	Albumin	2.6 g/dL	3.4-5.4 g/dL
	Total bilirubin	10.6 mg/dL	0.1-1.2 mg/dL
	Direct bilirubin	6.6 mg/dL	0.1-0.3 mg/dL
	Indirect bilirubin	4.2 mg/dL	0.2-0.8 mg/dL
	Aspartate aminotransferase	113 U/L	8-33 U/L
	Alanine aminotransferase	80 U/L	4-26 U/L
	Lipase	566 U/L	10-140 U/L
	Creatinine kinase	2501 U/L	55-170 U/L
Complete blood count	White blood cell count	13.2 x 10^3^ microLiter	4.5-11.0 x 10^3^ microLiter
	Platelet count	94 x 10^3^ microLiter	165-415 x 10^3^ microLiter
	Neutrophils %	85.70%	30-75%

Initial radiology workup included a computer tomography (CT) of the head with no acute intracranial abnormalities, ultrasound of the abdomen with findings of an unremarkable gallbladder and mild hepatic steatosis, renal ultrasound with no significant abnormality, and chest X-ray showing atelectasis in the left lung base. Weil's disease was clinically suspected based on the patient's travel history. Leptospirosis serology was ordered, but it didn't result yet. He was empirically started on ceftriaxone 2g intravenous every 24 hours and doxycycline 100mg intravenous twice a day. The patient was also observed for any possible Jarisch-Herxheimer reaction following the above-mentioned treatment. CT abdomen and pelvis without contrast, which showed no significant or acute abnormalities. Further workup included serology testing for Zika, dengue, West Nile, HIV, hepatitis, legionella, syphilis, tularemia, Bartonella, tick-borne illnesses, and histoplasmosis, all of which were non-reactive, along with a negative peripheral smear of malaria. On day five of the hospital course, the patient's clinical presentation was improved, and he was discharged on oral cefpodoxime 200 mg and doxycycline 100 mg twice a day for a total of nine days. He was told to follow up in the Infectious Disease clinic in two weeks. Upon follow-up in the clinic, he tested positive for leptospirosis IgM ab with continued overall improved symptomatology.

## Discussion

We present a rare case of Weil's disease - a complication of leptospirosis in a 43-year-old African American male presenting with sepsis, acute renal failure, and transaminitis diagnosed in the United States. Leptospirosis was initially reported in 1866 by Adolph Weil as a feverish condition accompanied by jaundice, enlarged spleen, kidney failure, and inflammation of the eyes in individuals who had contact with water. As a result, the severe form of the disease was named after him - Weil's disease [1,6]. In 1907, the organism *Spirochaeta interrogans, *the causative organism behind leptospirosis, was officially named based on its appearance under the microscope, which resembles a question mark shape [1]. Leptospirosis can be difficult to control because it can spread rapidly through the kidneys' proximal tubule without showing any symptoms or causing illness in the host rodent. The bacteria is eventually released in the urine, but in some cases, it can affect the kidneys by targeting the tubular lumen, which is the most common initial site of infection [7,8]. If left untreated or misdiagnosed, then the infection can progress to a severe form known as WD.

In WD, patients can have a severe cytokine storm due to increased levels of Interleukin-6, tumor necrosis factor-alpha, and Interleukin-10 which can cause multiple organ failure [3, 7]. Although the precise pathological process involved in WD is not yet fully comprehended, it has been observed that complications arise due to endothelial cell injury and vasculitis caused by endothelial edema, necrosis, and lymphocytic filtration [7]. Furthermore, WD, apart from affecting the kidneys, can also harm other organs like the gastrointestinal tract, lungs, liver, and pancreas [4]. Acute Kidney injury is the most common in the form of acute tubular necrosis resulting from conditions such as ischemia, sepsis, or septic shock [7]. This condition can also impact liver function and lead to jaundice. It can also cause respiratory issues such as pulmonary edema and acute respiratory distress syndrome [7]. Some case reports have mentioned acute pancreatitis being found in WD and a higher risk of bleeding due to the inflammation resulting in cutaneous and visceral hemorrhages [7, 9]. In our case, the patient had several of the above complications. Prompt diagnosis of his condition and adequate treatment significantly decreased his risk of deterioration.

Additionally, due to the presence of scleral icterus and transaminitis, the diagnosis of hepatitis was at the top of differential diagnosis as that is more common in the United States. Even though our patient was a classic presentation of WD, it is very rare to diagnose leptospirosis in the United States. There have been several case reports of leptospirosis in countries like Sri Lanka, Fiji, Colombia, and several other countries, but our case is one of the few ones to diagnose it in the United States [10,11,12].

## Conclusions

Leptospirosis is a rare disease and early recognition and treatment, especially in patients with a recent travel history, is essential in mitigating serious outcomes. Leptospirosis should be on the differential diagnosis list of travel-related infections. Prompt treatment can decrease morbidity and mortality related to Weil's disease.

## References

[1] Rajapakse S (2022). Leptospirosis: clinical aspects. Clin Med (Lond).

[2] (2023). Nationally notifiable infectious diseases and conditions, United States: weekly tables. https://wonder.cdc.gov/nndss/static/2023/07/2023-07-table800.html.

[3] Haake DA, Levett PN (2015). Leptospirosis in humans. Curr Top Microbiol Immunol.

[4] De Brito T, Silva AM, Abreu PA (2018). Pathology and pathogenesis of human leptospirosis: a commented review. Rev Inst Med Trop Sao Paulo.

[5] Ansdell V (2022). Leptospirosis. Netter's Infectious Diseases, Second Edition.

[6] Karpagam KB, Ganesh B (2020). Leptospirosis: a neglected tropical zoonotic infection of public health importance-an updated review. Eur J Clin Microbiol Infect Dis.

[7] Petakh P, Isevych V, Kamyshnyi A, Oksenych V (2022). Weil's disease-immunopathogenesis, multiple organ failure, and potential role of gut microbiota. Biomolecules.

[8] Marshall RB (1976). The route of entry of leptospires into the kidney tubule. J Med Microbiol.

[9] Hutajulu RB, Bramantono B, Rusli M, Arifijanto MV, Hadi U (2023). A rare case of Weil's disease with acute pancreatitis and acute kidney injury: focus on management - a case report. Ann Med Surg (Lond).

[10] Herath NJ, Kamburapola CJ, Agampodi SB (2016). Severe leptospirosis and pancreatitis; a case series from a leptospirosis outbreak in Anuradhapura district, Sri Lanka. BMC Infect Dis.

[11] Mayfield HJ, Smith CS, Lowry JH (2018). Predictive risk mapping of an environmentally-driven infectious disease using spatial Bayesian networks: a case study of leptospirosis in Fiji. PLoS Negl Trop Dis.

[12] Conroy AL, Gélvez M, Hawkes M, Rajwans N, Liles WC, Villar-Centeno LA, Kain KC (2014). Host biomarkers distinguish dengue from leptospirosis in Colombia: a case-control study. BMC Infect Dis.

